# Rapid sample processing for intracellular metabolite studies in *Penicillium ochrochloron* CBS 123.824: the FiltRes-device combines cold filtration of methanol quenched biomass with resuspension in extraction solution

**DOI:** 10.1186/s40064-016-2649-8

**Published:** 2016-07-04

**Authors:** Pamela Vrabl, Desiree J. Artmann, Christoph W. Schinagl, Wolfgang Burgstaller

**Affiliations:** Institute of Microbiology, University of Innsbruck, Technikerstrasse 25, 6020 Innsbruck, Austria

**Keywords:** *Penicillium ochrochloron*, Chemostat, Rapid biomass separation and resuspension technique, Cold-filtration, Energy charge, Nucleotides

## Abstract

**Background:**

Many issues concerning sample processing for intracellular metabolite studies in filamentous fungi still need to be solved, e.g. how to reduce the contact time of the biomass to the quenching solution in order to minimize metabolite leakage. Since the required time to separate the biomass from the quenching solution determines the contact time, speeding up this step is thus of utmost interest. Recently, separation approaches based on cold-filtration were introduced as promising alternative to cold-centrifugation, which exhibit considerably reduced contact times. In previous works we were unable to obtain a compact pellet from cold methanol quenched samples of the filamentous fungus *Penicillium ochrochloron* CBS 123.824 via centrifugation. Therefore our aim was to establish for this organism a separation technique based on cold-filtration to determine intracellular levels of a selected set of nucleotides.

**Results:**

We developed a cold-filtration based technique as part of our effort to revise the entire sample processing method and analytical procedure. The Filtration-Resuspension (FiltRes) device combined in a single apparatus (1) a rapid cold-filtration and (2) a rapid resuspension of the biomass in hot extraction solution. Unique to this is the injection of the extraction solution from below the membrane filter (FiltRes-principle). This caused the mycelial cake to detach completely from the filter membrane and to float upwards so that the biomass could easily be transferred into preheated tubes for metabolite extraction. The total contact time of glucose-limited chemostat mycelium to the quenching solution could be reduced to 15.7 ± 2.5 s, whereby each washing step added another 10–15 s. We evaluated critical steps like filtration time, temperature profile, reproducibility of results, and using the energy charge (EC) as a criterion, effectiveness of enzyme destruction during the transition in sample temperature from cold to hot. As control we used total broth samples quenched in hot ethanol. Averaged over all samples an EC of 0.93 ± 0.020 was determined with the FiltRes-principle compared to 0.89 ± 0.049 with heat stopped total broth samples.

**Conclusions:**

We concluded that for *P. ochrochloron* this technique is a reliable sample processing method for intracellular metabolite analysis, which might offer also other possible applications.

**Electronic supplementary material:**

The online version of this article (doi:10.1186/s40064-016-2649-8) contains supplementary material, which is available to authorized users.

## Background

Metabolic engineering of microorganism is a promising field of research which offers the possibility to produce chemical building-blocks and desired substances of interest from renewable resources (Lee et al. 2012). One prerequisite to turn microbes into highly effective cell-factories is an in-depth understanding of the physiological network, which in turn is strongly dependent on the reliability of the methods used to gain the metabolic data for modeling (Taymaz-Nikerel et al. 2009; Villas-Bôas et al. 2005). However, in contrast to the recent advances in analytical techniques, there are still some critical issues to be solved concerning sample processing (Ryan and Robards 2006; van Gulik et al. 2013; Villas-Bôas et al. 2005).

One major issue is that there is currently no—and probably never will be—standard protocol which is valid for all organisms and metabolites. On the contrary, several authors highlighted the need to adjust sample processing methods to the targeted metabolites and organisms (e.g. Nielsen and Jewett 2007; van Gulik et al. 2013; Villas-Bôas et al. 2005). To complicate matters further, this methodological adaptation might even be necessary on a phenotypic level since changes in physiology, plasma membrane and cell wall composition can affect the organism’s response to the applied methods (da Luz et al. 2014; van Gulik et al. 2013; Zakhartsev et al. 2015). These findings led to a series of critical evaluations and improvements of techniques for rapid sampling, quenching, separation of biomass, extraction of metabolites and evaporation (e.g. Bolten and Wittmann 2008; de Jonge et al. 2012; Douma et al. 2010; Schaub et al. 2006; Villas-Bôas et al. 2005; Zakhartsev et al. 2015).

Especially the quenching step, which is commonly performed with a −40 °C aqueous methanol solution, and the subsequent biomass separation via cold centrifugation (Mashego et al. 2007; van Gulik 2010), have proven to be critical in terms of metabolite loss (van Gulik et al. 2013), sample carryover (Douma et al. 2010) or salt precipitation (Zakhartsev et al. 2015). How to minimize this ‘cold-induced leakage of metabolites’ (da Luz et al. 2014) is a widely debated issue in metabolic studies and, considering the problem of organism specificity (van Gulik et al. 2013), not one which can be answered straightforwardly. Thus the method of choice to prevent metabolite leakage has to consider—besides organism specific issues—several factors such as the targeted metabolites (e.g. small molecules leak faster out of the cell than large molecules; Canelas et al. 2008), the chemical properties of the quenching solution (Bolten and Wittmann 2008; de Jonge et al. 2012; Villas-Bôas and Bruheim 2007), the quenching temperature (Canelas et al. 2008) and the total contact time to the quenching solution (Canelas et al. 2008; Villas-Bôas et al. 2005).

Since the total contact time with the quenching solution is strongly determined by the time required for the separation of the biomass from the quenching solution, one strategy to minimize metabolite leakage is to speed up the biomass separation step. Centrifugation is widely used (Mashego et al. 2007) because it is relatively simple and it is possible to process several samples simultaneously. Nevertheless, its limits can become critical for the experimental setup. For example, the minimum necessary centrifugation time relies on the formation of a compact pellet and typically ranges between 5 and 20 min (Villas-Bôas et al. 2005; Zakhartsev et al. 2015). If the centrifugation process has to be repeated because washing steps are needed, the increased total contact time may cause extensive metabolite leakage (van Gulik et al. 2013).

In some cases separating the combined quenching solution/culture broth with centrifugation can pose a severe challenge or is simply not possible. Nasution et al. (2006) reported that for glucose-limited chemostat mycelium of *Penicillium chrysogenum* it was necessary to use a swing-out rotor to obtain a compact pellet. Using centrifugation as separation technique for methanol-quenched chemostat mycelium of *Aspergillus niger* was not successful because no compact pellet was formed (Lameiras et al. 2015). Similarly, in a previous study of our work group all attempts failed to separate methanol-quenched glucose-limited chemostat mycelium of *Penicillium ochrochloron* with centrifugation from the quenching solution (Ganzera et al. 2006). In preliminary experiments for this study we used a swing-out rotor as suggested by Nasution et al. (2006). Although this somewhat improved the formation of a pellet, it was impossible to completely remove the supernatant in the subsequent decantation step—an issue which has been observed by others before and can lead to a severe overestimation of metabolite levels because of sample carry-over (Douma et al. 2010; Zakhartsev et al. 2015). Furthermore, the time needed to obtain a reasonable stable pellet by centrifugation was about 20 min and therefore distinctly exceeded the recommended maximum of 5 min (Zakhartsev et al. 2015). As many of our future targeted experimental conditions will require at least one washing step to remove extracellular metabolites, the total contact time to the quenching solution would thus increase to 40 min and more. Like in other organisms (Canelas et al. 2008), preliminary experiments with *P. ochrochloron* indicated a significant loss of metabolites within this time frame (Additional file 1: Fig S2).

So far, there are only a few alternative methods to centrifugation available. In the last years filtration based separation techniques have received more and more attention. Currently two major principles of filtration based techniques have been developed. With techniques based on ‘fast-filtration’ the quenching follows the rapid separation of biomass from the culture broth (da Luz et al. 2014). One drawback of this method is that it is not suitable for metabolites with a high turnover like ATP (Wittmann et al. 2004). With techniques based on ‘cold-filtration’, which have been introduced recently (Douma et al. 2010; Meinert et al. 2013; Lameiras et al. 2015), the sample is quenched first with cold methanol and then filtrated. If depth filters—like glass fibre filters—are used, then these filters have to be extracted together with the mycelial cake before it is removed by a centrifugation step from the extraction broth (Douma et al. 2010; Lameiras et al. 2015). Although filtration is comparably laborious and thus usually accompanied with a lower sampling frequency, there are some considerable advantages such as a more efficient removal of extracellular metabolites or a minimized risk for metabolite leakage because of the considerably reduced total contact times to the quenching solution (Douma et al. 2010).

One focus of our ongoing research project is to explore the intracellular concentration of a selected set of nucleotides in various physiological conditions (e.g. different nutrient limitations, different growth phases, different degree of organic acid excretion). Of special interest for our studies is the Energy Charge (Atkinson 1968), which is an important marker for the metabolic energy status. Furthermore, since turnover time of adenine nucleotides is among the shortest of all cellular metabolites (<1 s), this parameter is an extremely sensitive indicator whether or not the metabolic stop and the subsequent sample processing could keep metabolic activities halted (Faijes et al. 2007). As a prerequisite for this aim, our previous sample processing and analytical method (described in Ganzera et al. 2006) had to be considerably modified. This involved two subtasks: First, to revise the existing sample extraction protocols and to expand the analytical procedures to cover a broader range of targeted metabolites. The second subtask aimed at a complete revision of our general sample processing strategy, i.e. exchanging the established total broth method using a heat stop (described in Ganzera et al. 2006) in favor of a sequential sample processing method which involves a cold quenching step. To achieve this, we had to overcome one major obstacle, namely to solve our aforementioned difficulties with centrifugation of methanol quenched samples. Since filtration based separations have been reported to be superior concerning metabolite leakage (Douma et al. 2010; van Gulik et al. 2013), we opted for a cold-filtration-based technique. Our efforts resulted in the development of the FiltRes-principle, which combines the cold-filtration step and the subsequent rapid resuspension of the mycelium in the hot extraction solution in one single device. The FiltRes-principle allowed us for the first time to efficiently separate the mycelial biomass of *P. ochrochloron* from the quenching solution while meeting the demands for sample processing of metabolites with a fast intracellular turnover.

## Methods

### Organism and preculture

All experiments were performed with *Penicillium ochrochloron* CBS 123.824 (in previous works *P. simplicissimum*; Vrabl et al. 2008). This strain was derived from a *P. ochrochloron* wild type strain (CBS 123.823) through years of passaging (Vrabl et al. 2012) and has been well characterized in terms of organic acid excretion (e.g. Franz et al. 1993; Gallmetzer and Burgstaller 2001; Vrabl et al. 2012). Ammonium limited precultures of *P. ochrochloron* were cultivated for standardized 72 ± 1 h (Schinagl et al. 2016) at 30 °C on a rotary shaker (350 rpm) in a 1 M HEPES-glucose medium (Gallmetzer et al. 1998; Schinagl et al. 2016) and used as inoculum for bioreactor cultivations.

### Bioreactor cultivation conditions and sampling

Batch and chemostat cultivations were either executed in a Biostat M, Biostat B (Braun/Sartorius, Germany) or a KLF 2000 (Bioengineering, Switzerland) bioreactor, basically following the procedures as described in Ganzera et al. (2006) and Vrabl et al. (2008) with minor modifications. The working volumes ranged from 1.8 L (Biostat M, KLF 2000) to 4 L (Biostat B). All chemostat cultures were grown at a specific growth rate (µ) of 0.1^−^h at 30 °C, an aeration rate of 0.56 vvm, a stirrer tip speed of 2.12 m s^−1^ and a pH of 7 (kept constant with 0.2 N NaOH). Volumetric control for the chemostat cultures was achieved by means of an electronic balance (Mettler Toledo) for the KLF 2000, by a lateral overflow device (Biostat M) or an automated sensor-driven pump (Biostat B). Two hours after inoculation with 100 mL (Biostat M, KLF 2000) or 200 mL (Biostat B) filamentous preculture, the feed pump was started (Schinagl et al. 2016). After four to five hydraulic residence times, the cultures reached stable steady state conditions. Steady states were regularly checked for homogenous morphology by light microscopy (oil immersion, phase contrast), constant biomass formation, respiration, organic acid excretion and residual nutrient concentrations (details see “Analytics” section). Bioreactor batch cultivations were performed as described earlier (Vrabl et al. 2012). Samples were withdrawn from the bioreactors either with a syringe or directly sprayed into the quenching solution using a compressed air driven sampling device (KLF 2000) or with aid of the slight overpressure inside the bioreactor (Biostat M, Biostat B).

### Bioreactor media

Method development for the FiltRes-principle was carried out in a series of glucose-limited chemostat cultivations (Table 1). The FiltRes-principle was later tested also for other cultivation conditions, i.e. ammonium and phosphate-limited chemostat conditions, as well as for phosphate-limited batch cultivations. Media for chemostat cultivations were based on slightly modified media of previous works (Gallmetzer and Burgstaller 2002; Vrabl et al. 2008) and were prepared as described in Vrabl et al. (2008). Glucose-limited conditions (mM): glucose 1H_2_O (20), (NH_4_)_2_SO_4_ (12.5), KH_2_PO_4_ (5.8), MgSO_4_ 7H_2_O (1.6). Ammonium-limited conditions (mM): glucose 1H_2_O (200), (NH_4_)_2_SO_4_ (2), KH_2_PO_4_ (5.8), MgSO_4_ 7H_2_O (1.6). Phosphate-limited conditions (mM): glucose 1H_2_O (200), (NH_4_)_2_SO_4_ (6), KH_2_PO_4_ (0.11), MgSO_4_ 7H_2_O (1.6), KCl (5.91). Antifoam agent (Clerol FBA 5075, Germany) reduced from 0.1 % (w/v) to 0.01 % (for glucose and ammonium limitation) or 0.02 % (phosphate limitation) final concentration, respectively. For all three limitations 10 mL trace element solution were added per liter medium (preparation and composition as described in Vrabl et al. 2008). The medium for phosphate-limited batch cultivations was as described in Vrabl et al. (2012).

**Table 1:** Overview of performed glucose-limited chemostat cultivations (µ = 0.1^−h^, 30 °C, pH 7), sampling time points, applied FiltRes-prototype and other variations in sample protocols in course of the method development

Chemostat	General		Variations in experimental protocol			
	Bioreactor	Sampling time point (h)	FiltRes prototype	Washing steps	Extraction solvent	Analytical procedure
I	BB	64^TB^	GL	0^b^	50 % (v/v) ethanol	IN
II	BM	116^TB^	GL	0^b^	50 % (v/v) ethanol	IN
III	BB	80^TB^	GL	0^b^, 1^b^, 2^a^ and 3^b^	50 % (v/v) ethanol	IM
			POM	1^c^	50 % (v/v) ethanol	IM
IV	BB	90	GL	0^e^	50 % (v/v) ethanol	IM
V	BM	53^TB^	M	0^a^	50 % (v/v) ethanol	IM
VI	BM	42	POM	0^b^ and 1^a^	50 % (v/v) ethanol	IM
		73	POM	0^b^	50 % (v/v) ethanol	IM
		88^TB^	POM	0^b^ and 1^b^	50 % (v/v) ethanol	IM
VII	BM	69	POM	1^b^	50 % (v/v) ethanol	IM
VIII	BM	47	POM	0^c^	Buffered 50 % (v/v) ethanol	F
		67	POM	0^d^	Buffered 50 % (v/v) ethanol	F
		92	POM	0^d^	Buffered 50 % (v/v) ethanol	F

*Bioreactor* (working volume): BM, Biostat M (1.8 L); BB, Biostat B (4 L)

*FiltRes*-*prototypes*: GL, prototype with GL 45 cap; M, prototype made of metal; POM, prototype made of polyoxymethylene (details see “Methods” and Additional file 1: Fig. S1)

*Analytical procedure*: IN, initial method after Ganzera et al. (2006); IM, intermediary method after Krüger (2013); F, final method after Krüger (2013), for details see “Methods”

*Number of samples*: ^a^ n = 2, ^b^ n = 3, ^c^ n = 4, ^d^ n = 5, ^e^ n = 6

^TB^ At these sampling time points also heat stopped total broth samples were taken as reference samples (details see “Methods”)

**Fig. 1:**
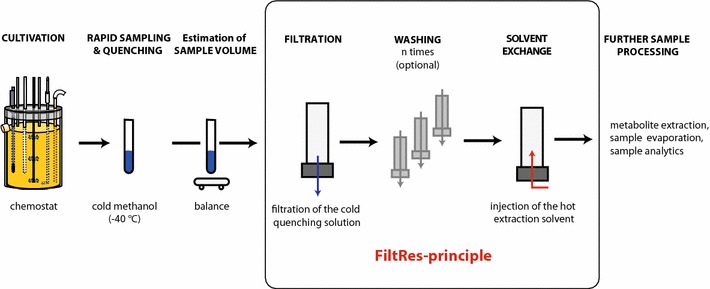
Experimental workflow of the sample processing using the FiltRes-device

**Fig. 2:**
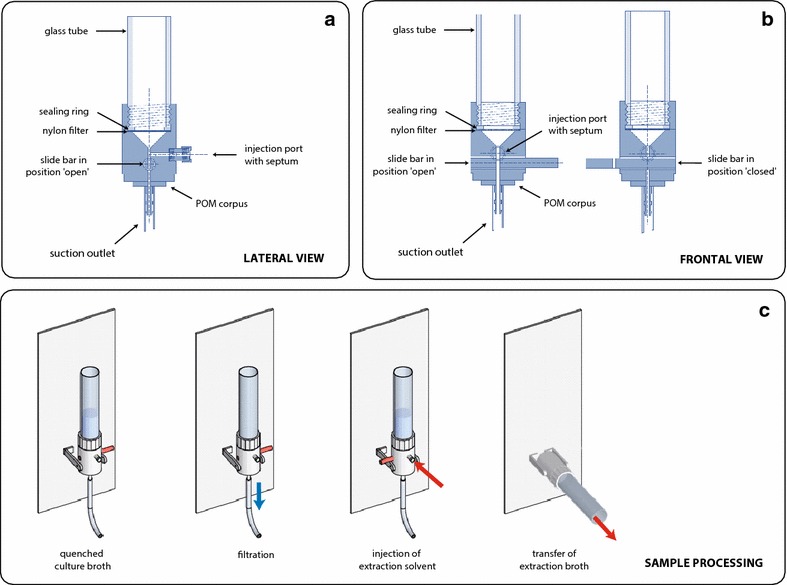
Sectional drawing of the FiltRes-device made of POM (**a**, **b**) and sample processing using the FiltRes-device (**c**) with the following steps: transfer of the quenched sample into the device and vacuum filtration (slide bar in open position). Injection of the extraction solution via the injection port (slide bar in closed position). Transfer of the extraction broth into preheated tubes for further extraction

**Fig. 3:**
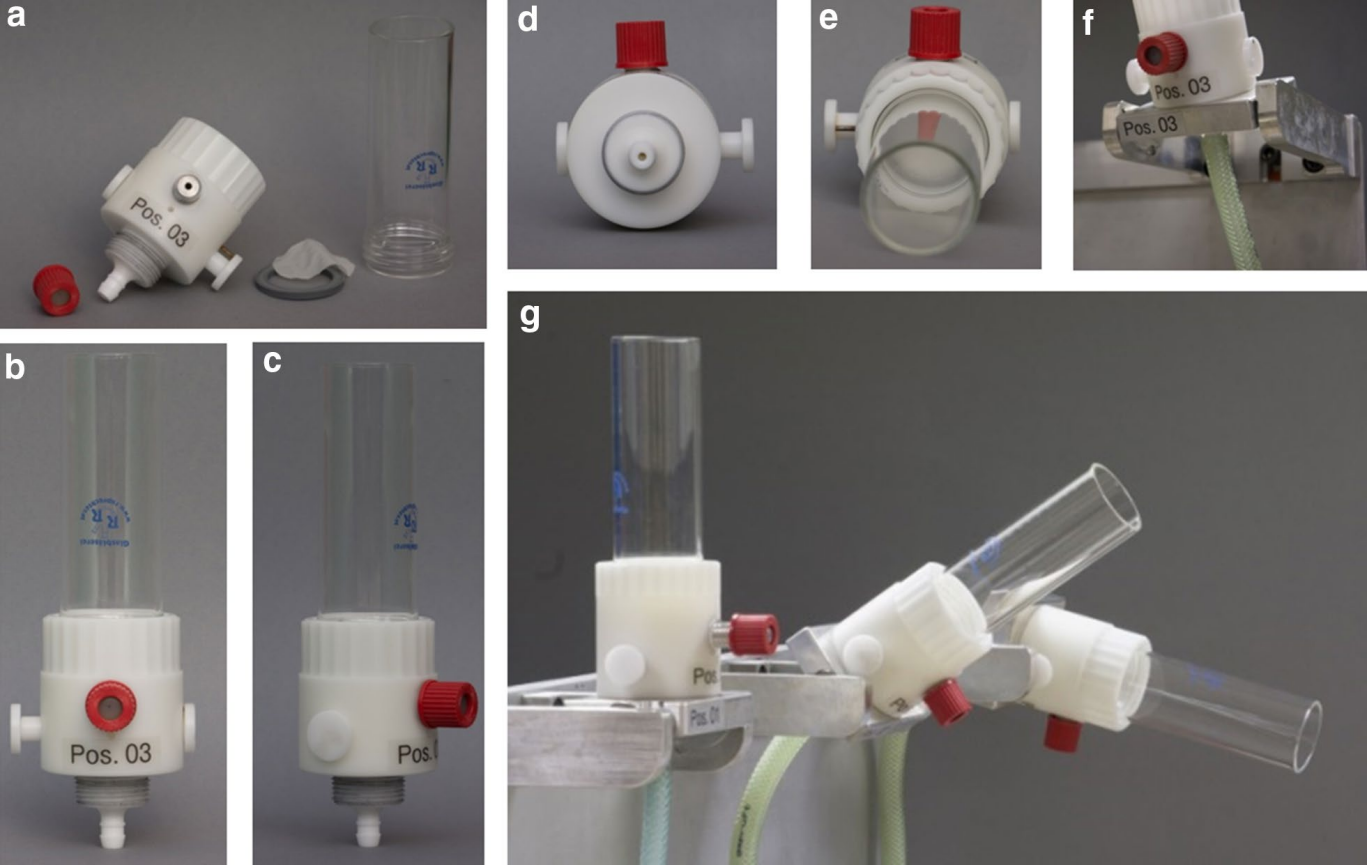
FiltRes-device made of POM. **a** disassembled into single parts, corpus with the slide bar still mounted, in **b** frontal, **c** lateral, **d** bottom and **e** top view, **f** mounted on a guide rail and in mid-tilted position and **g** arranged as quickly interchangeable triplets in untitled, mid-tiled and fully tilted position

The *FiltRes*-*principle* combines the cold-filtration step and the subsequent rapid resuspension of the resulting filter cake in the extraction solution in one single device (filtration and resuspension; FiltRes). Unique to this technique is the injection of the extraction solution from below the membrane filter, which causes the entire mycelial filter cake to detach from the filter and float upwards (details see “Sequential sample processing using the FiltRes-principle” section). This feature allows an easy transfer of the mixture of extraction solution and mycelium into preheated test tubes for further processing. In contrast to depth filter based methods (Douma et al. 2010, Lameiras et al. 2015), the FiltRes-principle offers the possibility to extract the mycelium apart from the filter. In the course of this work we developed several FiltRes-device prototypes (see Additional file 1: Fig. S1) using different materials and geometries to optimize the handling and to speed up the filtration and resuspension step. However, sample processing via the FiltRes-principle (Fig. 1, details see below) was similar for all prototypes and differed mainly in the way to close the suction outlet or to inject the hot extraction solution. For the final custom made device (Nerd-Toolz Inc., Austria; detailed schematics in Fig. 2a, b; photograph in Fig. 3) we selected polyoxymethylene (POM) as material for the corpus—a material which is known for its low abrasion, chemical stability and short term heat resistance of up to 150 °C (Moeller 2008). A nylon filter net (pore size 1 µm) was tightly fixed with a flat sealing gasket between the glass tube (GL 45 thread) and the POM-corpus. The suction outlet was opened or closed with a slide bar. The hot extraction solution was applied through a firmly sealed (two silicone septa) horizontal injection port (GL 18), which was connected to the upper part of the suction outlet above the slide bar. Every sealing and especially the septa of the injection port were regularly checked and renewed if needed. After each use all parts including the slide bar were dismantled to allow a thorough cleansing. If required, single fractions such as quenching or washing solutions could be collected by inserting a 50 mL Schott flask (Additional file 1: Fig. S3). The FiltRes-devices were mounted on a guard rail which enabled different tilting positions for facilitated transfer of the extraction broth into a polypropylene tube (Fig. 3f, g). To facilitate a quicker sequence of more than three samples, we machined a triple-bracket (Fig. 3g), i.e. three FiltRes-devices with their guard rails were arranged on a large single bracket, which could be rapidly replaced by another triple-bracket.

### Sequential sample processing using the FiltRes-principle

**Fig. 4:**
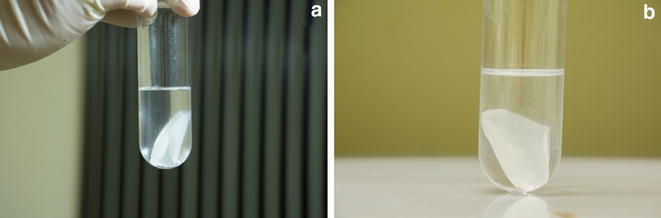
Filter cake of glucose-limited chemostat mycelium quenched in pure methanol and filtrated with the FiltRes-device. **a** Mycelial filter cake in the extraction solution after transfer into the tubes. **b** Still intact mycelial filter cake after rigorous shaking of the test tube. Note the difference to mycelium quenched in 60 % methanol, which quickly disperses in the extraction solution with the same treatment (illustrated in Additional file 2: Movie S1)

*Sequential sample processing using the FiltRes*-*principle* was as follows (sample processing demonstrated in Figs. 1, 2, 3 and Additional file 2: Movie S1 with the final prototype made out of POM): Prior to the experiment either 25 mL of 60 % (v/v) aqueous methanol or pure methanol were filled into 50 mL centrifuge tubes with screw caps (Nalgene, UK; polypropylene or polycarbonate). Then the tubes with the quenching solution were weighed and cooled down to −40 °C in a cooling bath (Fryka KB 18–40, Germany). As preliminary experiments indicated that quenching in pure methanol was problematic concerning the resulting filter cake (Fig. 4; details see “Results and discussion”) and leakage of organic acids (Additional file 1: Fig S2), 60 % methanol was used for all further experiments as quenching solution. Before the experiments were started, a modified dispenser [i.e. shortened discharge tube attached with a canule (1.8 mm outer diameter × 60 mm)], which contained the extraction solution, was placed in a water bath at 90 °C. The extraction solution was either unbuffered 50 % (v/v) aqueous ethanol (Ganzera et al. 2006), or in later experiments, buffered with EPPS (10 mM final concentration, pH 8.6) 50 % (v/v) aqueous ethanol (Table 1), which proofed to be in some cases beneficial for metabolite stability and recovery (unpublished results).

Approximately 5 mL culture broth was sprayed rapidly into the 25 mL precooled quenching solution, vortexed and instantaneous weighed to estimate the exact volume of the sample. The resulting mixture was then immediately transferred into the FiltRes-device for filtration (vacuum already applied, slide bar in opened position). After the filtration was finished, the slide bar was closed. To test the influence of washing steps, the mycelial filter cake was then additionally washed 1–3 times with 25 mL cold (−40 °C) quenching solution. Afterwards, the extraction solution was injected as follows. The septa of the injection port were pierced with the canule of the dispenser and 16.5 mL of hot extraction solution was rapidly injected. The injection of the extraction solution from below the filter net forced the mycelial filter cake to completely detach from the nylon filter net (detached filter cake see Fig. 4). This exchange of the quenching and extraction solutions was performed within a few seconds as illustrated in Additional file 2: Movie S1, where the handling of the FiltRes-device was demonstrated with a preculture mycelium. In the next step, the FiltRes-device was tilted to transfer the extraction broth into pre-weighed polypropylene or polycarbonate tube with screw caps (50 mL, Nalgene, UK; preheated in a water bath at 90 °C). Due to the device’s dead-volume (extraction solution below the filter), some of the injected extraction solution (1–2 mL) could not be transferred into the extraction tubes. This proportion of extraction solution, however, never came into direct contact with the mycelial cake as it remained below the filter disk. Even if metabolites had diffused into this dead volume, the determined intracellular metabolite concentration would have been only minimally distorted. This shortcoming will be eliminated in future prototypes. In any case, to guarantee similar volumetric extraction conditions, the volume transferred from the FiltRes-device to the preheated test tube was determined by weighing and, if necessary, compensated with hot extraction solution. Afterwards, the samples were placed into a shaking water bath at 90 °C and extracted as described later. Since these extracts were subsequently evaporated and resuspended in a defined volume of buffer, the compensation with hot extraction solution had no dilution effect. Concentrations of intracellular nucleotides were normalized to biomass (“Calculations and data analysis” section).

For method validation, the temperature profile was monitored, i.e. the temperature was measured at sensitive steps during the processing of the samples: The temperature of the quenching solution was measured prior and after quenching and also during the filtration step. The temperature of the extraction broth was measured (1) directly after injection into the FiltRes-device, (2) after the tube with the extraction solvent was placed into the water bath (90 °C) and (3) 1 min after that. Furthermore the time required for filtration and the total contact time of the sample to the quenching solution was determined.

### Simultaneous processed samples

*Simultaneous processed samples (i.e. heat stopped total broth samples)* served as reference method, which was based on the method of Ganzera et al. (2006), i.e. 5 mL sample was quenched in 5 mL 50 % (v/v) aqueous ethanol (tubes placed in a water bath at 90 °C prior to the experiments). To obtain comparable extraction conditions as with the sequential processed samples via the FiltRes-principle, the volumetric ratio of sample to extraction solution was modified to 1 mL sample and 16.5 mL extraction solution. 50 mL centrifuge tubes with screw caps (Nalgene, UK; polypropylene or polycarbonate) were filled with the extraction solution, closed, weighed and then placed in a water bath at 90 °C. The tubes were capped to prevent evaporation of ethanol and opened right before the quenching. The quenched samples were vortexed quickly and weighed to estimate the sample volume. The capped tubes were then again placed into a shaking water bath (90 °C) for extraction. Further sample processing was carried out as described below.

*Extraction and further sample processing* to measure intracellular nucleoside and nucleotide concentrations was performed with a slightly modified procedure according to Ganzera et al. (2006). For extraction, samples were placed for 10 min in a shaking water bath at 90 °C and then cooled for another 10 min on ice before they were filtrated (0.2 µm, Minisart SRP 15, Germany). Sample extracts were frozen at −70 to −80 °C, lyophilized (Het Power Dry PL6000, Thermo Fischer scientific, Austria) and then stored at −20 °C. Prior to analysis, the lyophilized samples were resuspended in 1 mL Tris–HCl buffer (50 mM, pH 8.5) and sonicated. Samples were then passed through a combined membrane filter (0.2 µm, Minisart SRP 15, Germany) and SPE extraction cartridge (Oasis HLB 6 cc, Waters, USA), and finally analyzed with HPLC and CE as described below.

### Analytics

Gravimetric estimation of dry weight, photometric determination of the residual nutrient concentrations of glucose, ammonium and phosphate, and estimation of organic acid concentrations via HPLC using a Bio-Rad Aminex HPX-87H column were carried out as described elsewhere (Vrabl et al. 2012). Homogenous mycelial morphology was checked for potential aberrations with light microscopy (phase contrast, magnification 500–1200 times with oil immersion). Dissolved oxygen tension in the bioreactor cultures was followed with a polarographic oxygen sensor (Mettler-Toledo/Ingold, Germany). The consumption of oxygen and the evolution of carbon dioxide were quasi-online monitored in the exhaust air of the bioreactor. For this the exhaust air was dried by a moisture exchanger (Perma Pur, NJ, USA) prior to the analysis with a respirometer (Biometric Systems, Germany).

As mentioned before, this study was part of our attempt to revise the sample processing and analytical procedure for nucleotides in *P. ochrochloron*. Thus, in course of this work also the analytical procedure was fine-tuned to the sample processing procedure and expanded by additional metabolites. All data for concentrations of intracellular nucleosides and nucleotides presented in this work stem from three different analytical procedures (Table 1): Starting point was our previously established gradient ion-pair HPLC method (Ganzera et al. 2006) for AMP, ADP, ATP, NAD and NADH using a Luna 5-µm C-8 column (Phenomenex, USA) and 50 mM aqueous TEA buffer and acetonitrile as mobile phase. This gradient HPLC-method was considerably revised and extended to include additional metabolites such as GTP, Inosine or IMP (in this work also referred to as ‘intermediary’ and ‘final’ methods, both described in detail in Krüger 2013). To double check metabolite identity and to quantify peaks which were not satisfyingly separated by the HPLC method-caused for instance by sample interference-capillary electrophoresis was chosen as a second analytics platform (Krüger 2013). With the optimized final method (Krüger 2013) each sample was analyzed twice: First, the samples were analyzed with ion-pair liquid chromatography on a Luna 5-µm C-8 column (Phenomenex, USA) and 5 mM aqueous DBA and acetonitrile as mobile phase. For quantification a fluorescence detector, which allowed the detection of low amounts of NADH and NADPH, and a diode array were used. Secondly, samples were also analyzed with capillary electrophoresis using a fused silica capillary (inner diameter 50 µm, effective length 620 mm) and 60 mM citric acid and 0.8 mM CTAB at pH 4.2 as buffer.

### Calculations and data analysis

Intracellular nucleotide concentrations were calculated based on the ratio of 1.3 mL intracellular water per gram dry weight (Firler et al. 1998). As proposed by Atkinson (1968) Energy Charge levels were calculated using the formula (ATP + 0.5 ADP)/(ATP + ADP + AMP). All Calculations were performed either with the software Excel 2010 from Microsoft or OriginPro 8 from Origin Lab. Data were tested for normal distribution with the Shapiro Wilks test (OriginPro 8). Figures and Tables were created using Microsoft Word, OriginPro 8 and Adobe Illustrator CS2.

## Results and discussion

### The FiltRes-principle: a new cold-filtration based separation technique

In this study we aimed at developing an alternative separation technique to centrifugation for *P. ochrochloron.* This technique should allow us for the first time to efficiently separate cold methanol quenched mycelium from the quenching solution while accounting for the fast intracellular turnover of nucleotides. Several prototypes (Additional file 1: Fig. S1) based on the FiltRes-principle were developed and tested (Table 1), which differed mainly in the materials used, the geometry and the way of handling. The final prototype made out of POM provided a considerably facilitated handling throughout the whole sample processing. Nevertheless, as we demonstrate later, the FiltRes-principle as such proved to be very robust over all tested prototypes. However, compared to decades of experience in cold-centrifugation techniques, cold-filtration techniques are still new to this field of research and many aspects concerning sample processing with these approaches remain yet to be explored and optimized. We thus also focus in this work on observations, of which some indicate—supported with recent findings in literature—that at least for our purposes methanol as quenching solution might have to be critically reconsidered.

### Applying the FiltRes-principle to glucose-limited chemostat grown *Penicillium ochrochloron*

To develop and improve the FiltRes-principle we used a series of independent glucose-limited chemostat cultures. This highly standardized cultivation conditions provided a reproducible homogenous sample material in terms of constant morphology and biomass concentrations or physiology, which facilitated a general assessment of the FiltRes-device’s performance. The following parameters were monitored for this work and will be elaborated in more detail in the next sections: (1) the filtration time, because it is the main determinant for the total contact time to the quenching solution; (2) the filtration performance of various sample types; (3) the intracellular nucleotide concentrations over a range of independent cultivations to assess the reproducibility of the FiltRes-principle; (4) the temperature profile, because cold stop techniques do not irreversibly damage enzymes. Thus special attention has to be given during the subsequent sample processing steps like the transition to the hot extraction conditions to avoid an unintended reactivation of metabolic activities (Zakhartsev et al. 2015). This holds especially true for metabolites with a fast turnover such as nucleotides. Finally, we used (5) the energy charge (EC; calculated as (ATP + 0.5 ADP)/(ATP + ADP + AMP); Atkinson 1968) as indicator whether or not enzyme based metabolism was kept in an inactivated state during rapid sampling, quenching, separation and the transfer to the hot extraction conditions (Faijes et al. 2007).

#### Filtration time

Using the final FiltRes-prototype made of POM (Figs. 2a, b, 3) we were able to reduce the total contact time to the cold aqueous methanol quenching solution to 15.7 ± 2.5 s (without any washing step) for glucose-limited mycelium of *P. ochrochloron*. Each additional washing step added 10–15 s to the procedure. In comparison to centrifugation, which required in our case at least 20 min and did not result in a reasonable pellet, this was a considerable improvement. In addition, if three single FiltRes-devices were arranged in a triplet (Fig. 3g) it allowed to process three samples in 1 min with a skilled team. Also the triplet can be rapidly exchanged with another triplet for a quicker succession of samples, which will be necessary for intended future studies with this organism.

For fungi the filtration times achieved with the FiltRes-device for glucose-limited mycelium of *P. ochrochloron* are amongst the shortest reported total contact times of cold-filtration based methods, which are typically below 60–90 s including several washing steps (e.g. Carnicer et al. 2012; Douma et al. 2010; Meinert et al. 2013). Compared to centrifugation a shortened contact time to the quenching solution was reported to be highly beneficial in terms of a reduced metabolite leakage (Douma et al. 2010).

#### Filtration characteristics and observations encountered during sample processing of methanol quenched samples

Overall, we observed that for the *P. ochrochloron* strain used in this study two factors influenced sample processing via the FiltRes-principle. First, it was of relevance for the resulting mycelial filter cake whether the samples were quenched in cold aqueous 60 % (v/v) methanol or pure methanol: although in both cases the initial mycelial filter cake appeared similar as shown in Fig. 4a, the mycelial filter cake of samples quenched in pure methanol remained more or less intact even after rigorous vortexing (Fig. 4b). This was not the case with the mycelial filter cake of samples quenched in aqueous 60 % (v/v) methanol, which could be easily resuspended as demonstrated in Additional file 2: Movie S1. Furthermore, the mycelial filter cakes differed in the texture: while the mycelial filter cake of samples quenched in aqueous 60 % (v/v) methanol were comparable to mycelial filter cakes of untreated culture broth, the mycelial filter cakes of samples quenched in pure methanol were gelatinous and dried out. This difference was especially apparent, if the extraction solution was removed for controlling purposes. As we could not exclude (1) a lower extraction efficiency and (2) because we observed a more pronounced metabolite leakage in samples which were quenched in pure methanol (Additional file 1: Fig. S2), all further experiments were performed with aqueous 60 % methanol. This pronounced metabolite leakage at higher methanol concentrations was also reported for other filamentous fungi (de Jonge et al. 2012; Lameiras et al. 2015).

Second, the nutritional status of the culture seemed to be—directly or indirectly—of relevance for the filtration characteristics: While methanol quenched ammonium-limited chemostat mycelium of *P. ochrochloron* was as easy and fast to handle as glucose-limited chemostat mycelium, phosphate-limited chemostat mycelium clogged the filter massively so that it was difficult to detach the mycelial cake from the membrane filter. Also in the course of phosphate-limited batch cultivation, the filtration procedure became slow and problematic shortly after phosphate became depleted in the medium. Before and after this critical phase—and despite approximately considerably higher biomass concentrations in the late phase of cultivation (Vrabl et al. 2012)—filtration was unproblematic. The reason for this observation cannot solely be attributed to morphology or biomass concentration. In all different nutrient limited chemostats the mycelium was filamentous and the biomass concentration was similar, whereas in case of the batch cultivation, the filtration problems occurred only within a short time frame of a few hours as mentioned above.

This leads to the question, if differences in medium composition and/or excreted fungal metabolites in combination with the choice for methanol as quenching solution were responsible for filtration problems: A recent study of Zakhartsev et al. (2015) critically investigated the common sample processing work flow involving a methanol quenching step. Amongst other observations they were able to demonstrate fast and extensive salt precipitation of medium components and extracellular fungal metabolites as a result of a significantly decreased solubility of many compounds after the samples were quenched in cold methanol. Apart from the fact that methanol as quenching solution has to be critically reconsidered in this respect as it can severely falsify the obtained metabolite levels (Zakhartsev et al. 2015), it might offer one explanation for the observed differences in filtration characteristics.

Also of interest in this context is a fast-filtration approach performed by da Luz et al. (2014) with *Escherichia coli*, where the above mentioned methanol effect can be ruled out because the filtration was performed prior to the quenching step. The authors observed that filtration characteristics were depended on the nutritional status (da Luz et al. 2014): when growing exponentially, the cultures were easier and faster to filtrate than under nutrient starved conditions, which severely clogged the filter. As these observations could not be explained by the biomass concentration, the authors hypothesized that the observed difference was probably due to changes in morphology and suggested a careful testing and—if required—also adaptation of filtration techniques to each organism and each growth phase anew. In any case, it indicates that albeit very promising, many influential aspects of filtration based separation techniques have yet to be explored.

#### Intracellular nucleotide levels of glucose-limited chemostat mycelium

**Fig. 5:**
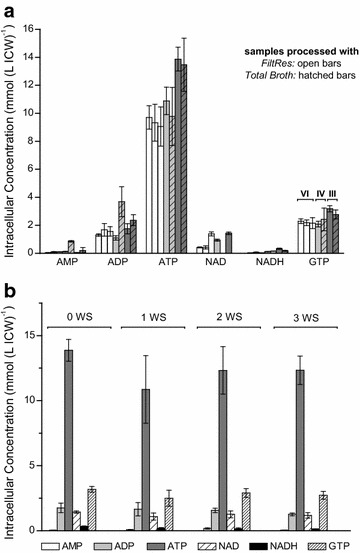
Intracellular nucleotide concentrations applying the FiltRes-principle on *Penicillium ochrochloron* CBS 123.824 grown under glucose-limited chemostat conditions at µ = 0.1 h^−1^. **a** Comparison of intracellular metabolite concentrations derived from samples with identical sample extraction protocol and analytical procedure (steady states from three independent chemostat cultivations; *light grey bars* chemostat III, *dark grey bars* chemostat IV, *white bars* chemostat VI at 42, 73 and 88 h). *Hatched bars* indicate data derived from heat stopped total broth samples (controls). Data are averages of at least three samples. Details to each chemostat see Table 1. Inosine and IMP were not detected. **b** Effect of washing steps (WS) with −40 °C cold 60 % (v/v) methanol on the intracellular metabolite concentrations. Inosine and IMP were not detected. Data were obtained from chemostat III (see Table 1) and are averages of three samples with exception of the samples with two washing steps, which were performed in duplicates

**Fig. 6:**
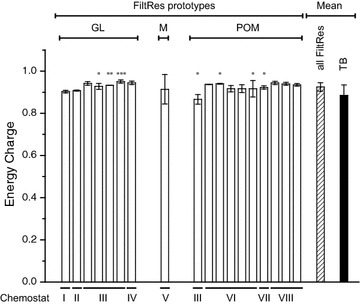
Energy charge of *Penicillium ochrochloron* CBS 123.824 grown under glucose-limited chemostat conditions at µ = 0.1 h^−1^ derived from eight independent chemostat cultivations with differing bioreactor types, FiltRes-prototypes (GL, M, POM; see section “Methods” and Additional file 1: Fig. S1), sample extraction and analytical procedures (details see Table 1). *Asterisks* mark the number of washing steps. Data are averages of three or more samples with the exception of III**, V and IV* which were performed in duplicate (see Table 1). *Hatched bar* average of all EC values derived from sample processing with the FiltRes-principle. *Black bar* average of all EC values derived from heat stopped total broth samples

Intracellular nucleotide levels, which were derived from a range of inter- and intra-cultivation samples and which were processed with different FiltRes-prototypes but the same quenching, extraction and analytical methods, were in the same order of magnitude (Fig. 5a). As these data were obtained via several different FiltRes-prototypes, it indicates that the FiltRes-principle per se was very robust for the investigated conditions and target metabolites. Additional washing steps did not significantly decrease intracellular nucleotide concentrations (Fig. 5b) nor affected the Energy Charge (Fig. 6; details see “Temperature profile and influence of the shift in sample temperatures on the energy charge” section). As any increase in metabolite leakage would have resulted in lower intracellular nucleotide levels in dependence of the number of washing steps, we conclude that repetitive washing steps with this cold-filtration based separation technique did not increase metabolite leakage at least for the targeted metabolites. In literature, washing steps are critically discussed with respect to metabolite leakage (e.g. Canelas et al. 2008, Douma et al. 2010). To our knowledge, there is only one study which compared the influence and efficiency of washing steps between a centrifugation based and a cold-filtration based approach. Douma and coworkers found for *P. chrysogenum* that the mode of separation was indirectly of high relevance. While washing steps caused metabolite leakage when samples were separated via centrifugation, this was not the case for cold filtrated samples. The authors concluded that this was most probably due to the significantly reduced total contact time to the quenching solution (Douma et al. 2010).

As indicated before, centrifugation with methanol quenched glucose-limited chemostat mycelium of *P. ochrochloron* was not possible and could thus not serve as control experiment for the FiltRes-principle. Thus, the presented data in this work are restricted to intracellular metabolite levels from cold methanol quenched samples and to heat stopped total broth samples (Ganzera et al. 2006) for comparison. Data of total broth samples (Fig. 5a hatched bars) were—except for NAD—in the same order of magnitude as data obtained with the FiltRes-principle. However, in two cultivations total broth samples showed a slight shift from ATP towards ADP and AMP, which resulted in a lower EC (see Additional file 1: Tables S1 and S2). This might indicate that the heat stop was less efficient in these cases.

Although attempted, we could not quantify metabolite leakage accurately with a differential approach (Douma et al. 2010), because the analysis of the quenching and washing solutions unexpectedly showed severe and irreversible issues with the HPLC-column (e.g. column clogging, sudden decline in column performance). As a consequence we were forced to replace the column several times. The reason for this is unclear. One possible explanation is that also in this case the aforementioned precipitation of medium compounds and metabolites in methanol plays a role. Such precipitates are known to be sometimes detrimental to the column performance or to cause other analytical problems (e.g. Nielsen and Larsen 2015, Zakhartsev et al. 2015). As we were unable to solve this problem during the course of this work, we did not yet make further attempts on this issue. However, it can be assumed from the data available in literature that metabolite leakage is most probably of minor relevance in our case: As already mentioned before, the considerably reduced contact time to the quenching solution obtained with cold-filtration is beneficial with respect to minimizing metabolite leakage (Douma et al. 2010). In addition, nucleotides—although also reported to leak from cells (e.g. Gonzalez et al. 1997)—generally leak to a smaller extent than smaller compounds such as organic acids because of their comparably large molecule size (Canelas et al. 2008).

#### Temperature profile and influence of the shift in sample temperatures on the energy charge

To assess if enzymatic activity was kept inactive during rapid sampling, quenching, filtration and the transfer to the hot extraction conditions, we monitored the sample temperature at several sensitive steps during sample processing and additionally used the energy charge (EC; Atkinson 1968) as indicator (Faijes et al. 2007).

After spraying the sample into the −40 °C cold quenching solution, the temperature of the resulting mixture rose to −18.6 ± 2.52 °C. During the short time of filtration the temperature always remained below −10 °C and the mycelial filter cake came into contact with the extraction solution within a few seconds as can be seen in Additional file 2: Movie S1. The injection of the hot extraction solution resulted in a drop in temperature of the extraction broth to 61.6 ± 0.23 °C, but quickly increased to the final extraction temperatures after the tubes were placed in the shaking water bath at 90 °C. The average EC of methanol quenched glucose-limited mycelium of *P. ochrochloron* CBS 123.824 calculated from a series of chemostat cultivations (Fig. 6) was 0.93 ± 0.020. In comparison, the obtained average EC-values with heat stopped total broth samples (method slightly modified after Ganzera et al. 2006) were in the range of 0.89 ± 0.049 (data represent means from a total of 20 samples from five independent cultivations (Table 1), see also Additional file 1: Table S1). Due to the higher scattering, these average EC values gained from the total broth method were not significantly lower as the average values obtained using the FiltRes-principle. Overall, these high EC values are typical for fungi under glucose-limited growth conditions (e.g. Nasution et al. 2006, Pitt and Bull 1982). Considering the FiltRes-principle it indicates that the metabolism was effectively halted during the whole sample processing.

Nevertheless, the critical transition in sample temperature from cold-quenching to hot extraction conditions deserves closer attention. This transition, which has rarely been addressed in literature so far, was identified by Zakhartsev et al. (2015) as sensitive spot for uncontrolled reactivation of enzymatic activity which in consequence can cause falsely estimated metabolite levels. While Zakhartsev et al. (2015) recommended this transition to occur in sub-second scale, it is apparent that this cannot be achieved with existing cold-filtration methods: There is inevitably a short period of time in the range of a few seconds which are necessary to transfer the mycelial filter cake into the hot extraction solution (e.g. Douma et al. 2010; present work Additional file 2: Movie S1). Whether or not the transition to hot extraction conditions is fast enough to avoid enzymatic reactivation, can only be indirectly assessed by parameters like the EC. A too slow metabolic quenching or an ineffective halt of enzymatic activity would have caused a drop in EC (Faijes et al. 2007; Ganzera et al. 2006). Thus the high EC values achieved in this study hint that neither the time needed for this transfer or the shift in temperature were critical in this respect. This is supported by other studies where similar high EC values were achieved from samples treated with cold stops followed by heat extraction (e.g. Nasution et al. 2006). However, this might not apply to other metabolic reactions and thus certainly necessitates further investigations.

## Conclusions

The purpose of this study was to overcome our previous difficulties to separate cold methanol quenched mycelium of *P. ochrochloron* via centrifugation from the quenching solution. Our efforts resulted in the development of the FiltRes-principle, which combines a cold-filtration based separation and the subsequent rapid resuspension of the resulting mycelial filter cake in the extraction solution in one single device (filtration and resuspension; FiltRes). Unique to this technique is the injection of the extraction solution from below the membrane filter, which causes the entire mycelial filter cake to detach from the filter and float upwards. This feature allowed an easy transfer of the mixture of extraction solution and mycelium into preheated test tubes for further processing. Using the FiltRes-principle enabled us for the first time to efficiently separate cold methanol quenched mycelium of *P. ochrochloron* from the quenching solution while also accounting for the fast intracellular turnover times of our targeted metabolites. The achieved total contact times for glucose-limited mycelium of *P. ochrochloron* were 15.7 ± 2.5 s (disregarding washing steps), which is amongst the shortest reported total contact times of cold-filtration based methods for fungi. Data derived with this method were reproducible. Furthermore, the high Energy Charge of 0.93 ± 0.020 indicated that enzymatic activity was effectively halted during the whole sample processing. We conclude that for *P.ochrochloron* this technique is a reliable sample processing method for intracellular metabolite analysis, which might offer also other possible applications. However, some of our finding indicated that at least for our purposes methanol as quenching solution might have to be critically reconsidered.

In addition, we applied the FiltRes-principle in our laboratory also in other experimental contexts which involved the filtration and resuspension of anaerobic fungi such as *Neocallimastix* sp. in different media (unpublished results). This suggested that the FiltRes-principle might be also useful for non-metabolomics related applications. We are aware that one hurdle to test novel applications is the availability of the respective construction at low costs. As especially the GL-prototype (Additional file 1: Fig. S1a) can be constructed out of materials which are typically available in laboratories or can be purchased cheaply, we decided to present the data of all prototypes. We hope that this facilitates testing the FiltRes-principle for other researcher if it is suitable for their purposes or if it could be modified to meet the demands for their applications and organisms.

## Additional files

10.1186/s40064-016-2649-8 Succession of FiltRes-prototypes used in this work. **Figure S2.** Metabolite Leakage in Penicillium ochrochloron CBS 123.824 in dependence of the methanol concentration and the contact time. **Figure S3.** Typical experimental setup aimed at estimating metabolite leakage with a differential approach. **Table S1.** Data set depicted in Fig. 6, levels of Energy Charge. **Table S2.** Data set depicted in Fig. 5a, b, intracellular nucleotide levels.
10.1186/s40064-016-2649-8 Applying the FiltRes-principle to methanol quenched preculture mycelium of *Penicillium ochrochloron*.

## Abbreviations

ADP: adenosine diphosphate
AMP: adenosine monophosphate
ATP: adenosine triphosphate
BB: Biostat B bioreactor
BM: Biostat M bioreactor
CTAB: cetyltrimethylammonium bromide
DBA: dibutyl amine
EC: energy charge, calculated as ([ATP] + 0.5[ADP)]/([ATP] + [ADP] + [AMP])
EPPS: 4-(2-hydroxyethyl)-1-piperazinepropanesulfonic acid
FiltRes-device: filtration resuspension device
GL: FiltRes-prototype using a GL-cap (Additional file 1: Fig. S1)
GTP: guanosine triphosphate
HEPES: 4-(2-hydroxyethyl)-1-piperazineethanesulfonic acid
I: inosine
IN, IM, F: initial, intermediary and final analytical protocol (described in “Methods”)
ICW: intracellular water
IMP: inosine monophosphate
M: FiltRes-prototype made of metal (Additional file 1: Fig. S1)
NAD: nicotinamide adenine dinucleotide
NADH: nicotinamide adenine dinucleotide reduced
n.d.: not detected
POM: polyoxymetylene
TB: total broth samples
TEA: triethyl amine
WS: washing steps
µ: specific growth rate

## References

[CR1] Atkinson DE (1968). The energy charge of the adenylate pool as a regulatory parameter: interaction with feedback modifiers. Biochemistry.

[CR2] Bolten CJ, Wittmann C (2008). Appropriate sampling for intracellular amino acid analysis in five phylogenetically different yeasts. Biotechnol Lett.

[CR3] Canelas AB, Ras C, ten Pierick A, van Dam JC, Heijnen JJ, van Gulik WM (2008). Leakage-free rapid quenching technique for yeast metabolomics. Metabolomics.

[CR4] Carnicer M, Canelas AB, ten Pierick A, Zeng Z, van Dam J, Albiol J, Ferrer P, Heijnen JJ, van Gulik WM (2012). Development of quantitative metabolomics for *Pichia pastoris*. Metabolomics.

[CR5] da Luz JA, Hans E, Zeng AP (2014). Automated fast filtration and on-filter quenching improve the intracellular metabolite analysis of microorganisms. Eng Life Sci.

[CR6] de Jonge LP, Douma RD, Heijnen JJ, van Gulik WM (2012). Optimization of cold methanol quenching for quantitative metabolomics of *Penicillium chrysogenum*. Metabolomics.

[CR7] Douma RD, de Jonge LP, Jonker CT, Seifar RM, Heijnen JJ, van Gulik WM (2010). Intracellular metabolite determination in the presence of extracellular abundance: application to the penicillin biosynthesis pathway in *Penicillium chrysogenum*. Biotechnol Bioeng.

[CR8] Faijes M, Mars AE, Smid EJ (2007). Comparison of quenching and extraction methodologies for metabolome analysis of *Lactobacillus plantarum*. Microb Cell Fact.

[CR9] Firler H, Gallmetzer M, Burgstaller W, Schinner F (1998). Citrate efflux in *Penicillium simplicissimum*: fundamental methods for the in vivo study of efflux kinetics. Food Technol Biotechnol.

[CR10] Franz A, Burgstaller W, Müller B, Schinner F (1993). Influence of medium components and metabolic inhibitors on citric acid production by *Penicillium simplicissimum*. J Gen Microbiol.

[CR11] Gallmetzer M, Burgstaller W (2001). Citrate efflux in glucose- limited and glucose- sufficient chemostat culture of *Penicillium simplicissimum*. Antonie Van Leeuwenhoek.

[CR12] Gallmetzer M, Burgstaller W (2002). Efflux of organic acids in *Penicillium simplicissimum* is an energy-spilling process, adjusting the catabolic carbon flow to the nutrient supply and the activity of catabolic pathways. Microbiology.

[CR13] Gallmetzer M, Müller B, Burgstaller W (1998). Net efflux of citrate in *Penicillium simplicissimum* is mediated by a transport protein. Arch Microbiol.

[CR14] Ganzera M, Vrabl P, Wörle E, Burgstaller W, Stuppner H (2006). Determination of adenine and pyridine nucleotides in glucose-limited chemostat cultures of *Penicillium simplicissimum* by one-step ethanol extraction and ion-pairing liquid chromatography. Anal Biochem.

[CR15] Gonzalez B, Francois J, Renaud M (1997). A rapid and reliable method for metabolite extraction in yeast using boiling buffered ethanol. Yeast.

[CR16] Krüger A (2013) Analysis of metabolites in plants and filamentous fungi, on the examples of *Oroxylum indicum* and *Penicillium ochrochloron*. Dissertation, University of Innsbruck

[CR17] Lameiras F, Heijnen JJ, van Gulik WM (2015). Development of tools for quantitative intracellular metabolomics of *Aspergillus niger* chemostat cultures. Metabolomics.

[CR18] Lee JW, Na D, Park JM, Lee J, Choi S, Lee SY (2012). Systems metabolic engineering of microorganisms for natural and non-natural chemicals. Nat Chem Biol.

[CR19] Mashego MR, Rumbold K, De Mey M, Vandamme E, Soetaert W, Heijnen JJ (2007). Microbial metabolomics: past, present and future methodologies. Biotechnol Lett.

[CR20] Meinert S, Rapp S, Schmitz K, Noack S, Kornfeld G, Hardiman T (2013). Quantitative quenching evaluation and direct intracellular metabolite analysis in *Penicillium chrysogenum*. Anal Biochem.

[CR21] Moeller E (2008). Handbuch Konstruktionswerkstoffe: Auswahl, Eigenschaften, Anwendung.

[CR22] Nasution U, van Gulik WM, Kleijn RJ, van Winden WA, Proell A, Heijnen JJ (2006). Measurement of intracellular metabolites of primary metabolism and adenine nucleotides in chemostat cultivated *Penicillium chrysogenum*. Biotechnol Bioeng.

[CR23] Nielsen J, Jewett MC, Nielsen J, Jewett MC (2007). The role of Metabolomics in systems biology. Metabolomics: a powerful tool in systems biology. Topics in current genetics series.

[CR24] Nielsen KF, Larsen TO (2015). The importance of mass spectrometric dereplication in fungal secondary metabolite analysis. Front Microbiol.

[CR25] Pitt DE, Bull AT (1982). The adenine nucleotide composition of growing and stressed cultures of *Trichoderma aureoviride*. Exp Mycol.

[CR26] Ryan D, Robards K (2006). Metabolomics: the greatest omics of them all?. Anal Chem.

[CR27] Schaub J, Schiesling C, Reuss M, Dauner M (2006). Integrated sampling procedure for metabolome analysis. Biotechnol Prog.

[CR28] Schinagl CW, Vrabl P, Burgstaller W (2016). Adapting high-resolution respirometry to glucose-limited mycelium of the filamentous fungus *Penicillium ochrochloron*: method development and standardization. PLoS ONE.

[CR29] Taymaz-Nikerel H, de Mey M, Ras C, ten Pierick A, Seifar RM, van Dam JC, Heijnen JJ, van Gulik WM (2009). Development and application of a differential method for reliable metabolome analysis in *Escherichia coli*. Anal Biochem.

[CR30] van Gulik WM (2010). Fast sampling for quantitative microbial metabolomics. Curr Opin Biotechnol.

[CR31] van Gulik WM, Canelas AB, Seifar RM, Heijnen JJ, Lämmerhofer M, Weckwerth W (2013). The sampling and sample preparation problem in microbial metabolomics. Metabolomics in practice: successful strategies to generate and analyze metabolic data.

[CR32] Villas-Bôas SG, Bruheim P (2007). Cold glycerol–saline: the promising quenching solution for accurate intracellular metabolite analysis of microbial cells. Anal Biochem.

[CR33] Villas-Bôas SG, Højer-Pedersen J, Åkesson M, Smedsgaard J, Nielsen J (2005). Global metabolite analysis of yeasts: evaluation of sample preparation methods. Yeast.

[CR34] Vrabl P, Mutschlechner W, Burgstaller W (2008). Characteristics of glucose uptake by glucose- and NH_4_-limited grown *Penicillium ochrochloron* at low, medium and high glucose concentration. Fungal Genet Biol.

[CR35] Vrabl P, Fuchs V, Schinagl CW, Burgstaller W (2012). Organic acid excretion in *Penicillium ochrochloron* increases with ambient pH. Front Microbiol.

[CR36] Wittmann C, Krömer JO, Kiefer P, Binz T, Heinzle E (2004). Impact of the cold shock phenomenon on quantification of intracellular metabolites in bacteria. Anal Biochem.

[CR37] Zakhartsev M, Vielhauer O, Horn T, Yang X, Reuss M (2015). Fast sampling for quantitative microbial metabolomics: new aspects on cold methanol quenching: metabolite co-precipitation. Metabolomics.

