# Supplementary material for: Rapid sample processing for intracellular metabolite studies in Penicillium ochrochloron CBS 123.824: the FiltRes-device combines cold filtration of methanol quenched biomass with resuspension in extraction solution
Source: Springerplus. 2016 Jul 4;5(1):966. doi: 10.1186/s40064-016-2649-8 (PMC4932030; doi:10.1186/s40064-016-2649-8)
Supplement: Supplementary file 1 — 10.1186/s40064-016-2649-8 Succession of FiltRes-prototypes used in this work. Figure S2. Metabolite Leakage in Penicillium ochrochloron CBS 123.824 in dependence of the methanol concentration and the contact time. Figure S3. Typical experimental setup aimed at estimating metabolite leakage with a differential approach. Table S1. Data set depicted in Fig. 6, levels of Energy Charge. Table S2. Data set depicted in Fig. 5a, b, intracellular nucleotide levels. [file 40064_2016_2649_MOESM1_ESM.docx]

**Additional file 1** to the original research article in the journal SpringerPlus:

**Rapid sample processing for intracellular metabolite studies in *Penicillium ochrochloron*CBS 123.824:**

**The FiltRes-device combines cold filtration of methanol quenched biomass with resuspension in extraction solution**

Pamela Vrabl*, Desiree J. Artmann, Christoph W. Schinagl and Wolfgang Burgstaller

University of Innsbruck, Institute of Microbiology, Technikerstrasse 25

6020 Innsbruck, Austria

* CORRESPONDING AUTHOR: Dr. Pamela Vrabl, University of Innsbruck

Institute of Microbiology, Technikerstrasse 25, A-6020 Innsbruck, Austria

Email: pamela.vrabl@uibk.ac.at, Tel.: +43-512-507-51241, Fax.: +43-512-507-2929


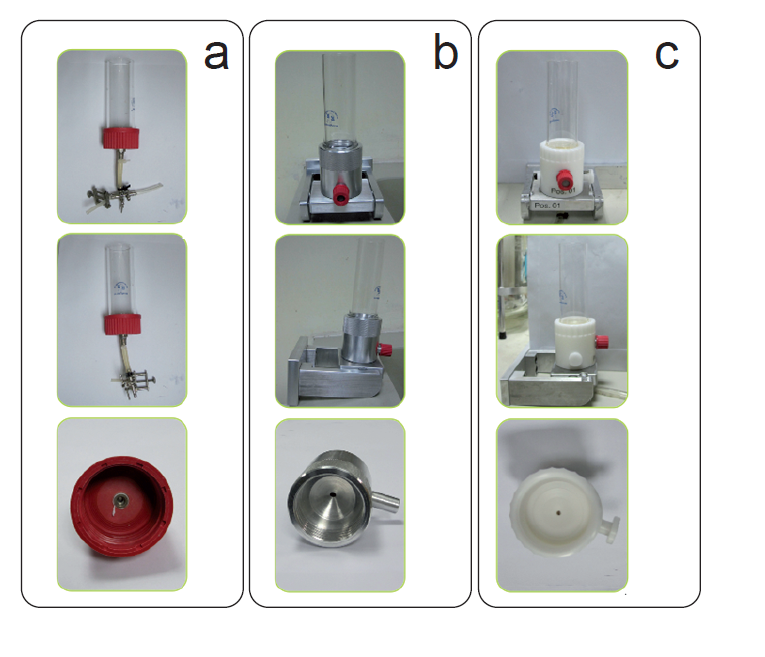


**Figure S1 Succession of FiltRes-prototypes used in this work.** (a) initial prototype using a GL 45 cap (in order from top to bottom) in frontal and lateral view and with dismantled bottom part in top view, (b) prototype out of metal (in order from top to bottom) in frontal and lateral view and with dismantled bottom part in top view, (c) final prototype made of POM (in order from top to bottom) in frontal and lateral view and with dismantled bottom part in top view (for more details see main manuscript, Fig. 2 and Fig. 3).

**Figure S2 Metabolite Leakage in *Penicillium ochrochloron*CBS 123.824 in dependence of the methanol concentration and the contact time.** (A) Series of preliminary experiments with methanol stop and metabolite extraction in HCl as described in Gallmetzer et al. (1998), Archives of Microbiology 169, 353-359. (B) Preliminary experiments with quenching in methanol, sample processing with the FiltRes-device and extraction in ethanol as described in the main manuscript. Following extraction, sample extracts were evaporated (Eppendorf concentrator 5301, Germany), dissolved in a defined volume of distilled water (either 1 or 2 mL), filtrated (0.2 µm cellulose acetate membrane filter, Sartorius, Germany) and then analyzed by HPLC (see section analytics main manuscript). n.d. … not detected.


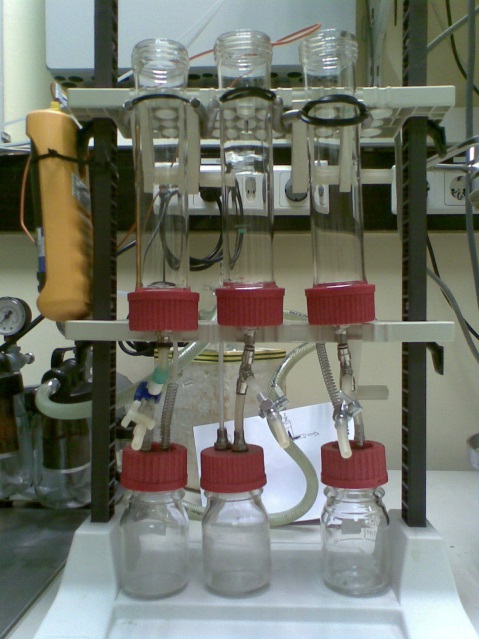


**Figure S3 Typical experimental setup aimed at estimating metabolite leakage with a differential approach.** Three FiltRes-devices (illustrated with GL-prototypes) were each equipped with an inserted 50 mL Schott-flasks to collect the filtrated quenching or washing solution.

**Table S1 Data set depicted in Fig. 6; levels of Energy Charge.** Details to the experimental setup of the single chemostats and to the prototypes see Tab. 1 main manuscript and additional file 1: Fig. S1. The energy charge (EC) was calculated using the formula (ATP + 0.5 ADP)/(ATP + ADP + AMP) as proposed by Atkinson (1968). The number of asterisks indicates the number of performed washing steps.

| **Chemostat** | **FiltRes prototype (GL, M or POM)**  **or total broth (TB)** | **EC** | **SD** | **Number of samples (n)** | **Comment** |
| --- | --- | --- | --- | --- | --- |
| I | GL | 0.90297 | ±0.00615 | 3 |  |
| I | TB | 0.83727 | ±0.01071 | 3 |  |
| II | GL | 0.90822 | ±0.00249 | 3 |  |
| II | TB | 0.92797 | ±0.03116 | 3 |  |
| III | GL | 0.94175 | ±0.00838 | 3 |  |
| III | TB | 0.92007 | ±0.02756 | 2 |  |
| *III | GL | 0.92804 | ±0.01384 | 3 |  |
| *III | POM | 0.86634 | ±0.02225 | 4 |  |
| **III | GL | 0.93256 | ±0.00016 | 2 |  |
| ***III | GL | 0.95058 | ±0.0075 | 3 |  |
| IV | GL | 0.94467 | ±0.00761 | 4 |  |
| IV | TB | 0.81053 | ±0.00720 | 2 |  |
| V | M | 0.91427 | ±0.06975 | 2 |  |
| V | TB | 0.89698 | ±0.03837 | 4 |  |
| VI (42 h) | POM | 0.93757 | ±0.00042 | 3 |  |
| *VI (42 h) | POM | 0.9402 | ±0.00179 | 2 |  |
| VI (73 h) | POM | 0.91686 | ±0.01533 | 3 |  |
| VI (88 h) | POM | 0.91707 | ±0.01838 | 3 |  |
| VI (88 h) | TB | 0.91889 | ±0.01875 | 6 | No biomass values available |
| *VI (88 h) | POM | 0.91673 | ±0.03858 | 3 |  |
| *VII | POM | 0.92254 | ±0.0082 | 3 |  |
| VIII (47 h) | POM | 0.94415 | ±0.00794 | 4 |  |
| VIII (67 h) | POM | 0.93999 | ±0.00701 | 5 |  |
| VIII (92 h) | POM | 0.93479 | ±0.00594 | 5 |  |

**Table S2 Data set depicted in Fig. 5a and 5b; intracellular nucleotide levels.** Details about the experimental setup of the single chemostats see Tab. 1 of the main manuscript. Normalization of data to mmol per litre intracellular water (ICW) was performed as described in the section Materials and Methods of the main manuscript. Abbreviations: WS … Washing steps of samples processed with the FiltRes device, TB … total broth samples, n.d. … not detected.

| Chemostat | **III** | | | | | | | | | | **IV** | | | |
| --- | --- | --- | --- | --- | --- | --- | --- | --- | --- | --- | --- | --- | --- | --- |
| WS or TB | **0 WS** |  | **1 WS** |  | **2 WS** |  | **3 WS** |  | **TB** |  | **0 WS** |  | **TB** |  |
| Metabolites | mmol  (L ICW)^-1^ | SD | mmol  (L ICW)^-1^ | SD | mmol  (L ICW)^-1^ | SD | mmol  (L ICW)^-1^ | SD | mmol  (L ICW)^-1^ | SD | mmol  (L ICW)^-1^ | SD | mmol  (L ICW)^-1^ | SD |
| **NAD** | 1.4277 | *±0.0897* | 1.0889 | *±0.2694* | 1.2584 | *±0.2610* | 1.1778 | *±0.2137* | n.d. |  | 0.9359 | *±0.0714* | n.d. |  |
| **NADH** | 0.3282 | *±0.0452* | 0.1963 | *±0.0425* | 0.1461 | *±0.0647* | 0.1253 | *±0.0155* | 0.1699 | *±0.0259* | 0.1172 | *±0.0174* | 0.1607 | *±0.0057* |
| **AMP** | 0.0385 | *±0.0071* | 0.0789 | *±0.0125* | 0.1673 | *±0.0504* | 0.0430 | *±0.0020* | 0.2096 | *±0.1947* | 0.1179 | *±0.0194* | 0.8608 | *±0.0514* |
| **ADP** | 1.7544 | *±0.3640* | 1.6514 | *±0.5107* | 1.5618 | *±0.1712* | 1.2548 | *±0.1111* | 2.3534 | *±0.3913* | 1.1001 | *±0.1561* | 3.6824 | *±1.0699* |
| **ATP** | 13.8680 | *±0.8467* | 10.8640 | *±2.6041* | 12.3334 | *±1.8293* | 12.3424 | *±1.0944* | 13.4548 | *±1.9010* | 10.8819 | *±0.9772* | 9.7771 | *±2.0787* |
| **GTP** | 3.1761 | *±0.2217* | 2.4945 | *±0.6221* | 2.8976 | *±0.3403* | 2.7224 | *±0.2989* | 2.7610 | *±0.3191* | 2.0903 | *±0.2043* | 2.4222 | *±0.8052* |
| **IMP** | n.d. |  | n.d. |  | n.d. |  | n.d. |  | n.d. |  | n.d. |  | n.d. |  |
| **Inosine** | n.d. |  | n.d. |  | n.d. |  | n.d. |  | n.d. |  | n.d. |  | n.d. |  |

| Chemostat | **VI** | | | | | |
| --- | --- | --- | --- | --- | --- | --- |
| WS or TB | **0 WS** |  | **0 WS** |  | **0 WS** |  |
| Metabolites | mmol  (L ICW)^-1^ | SD | mmol  (L ICW)^-1^ | SD | mmol  (L ICW)^-1^ | SD |
| **NAD** | 0.4213 | *±0.0373* | 0.4226 | *±0.1130* | 1.3845 | *±0.1433* |
| **NADH** | 0.0257 | *±0.0085* | 0.0693 | *±0.0280* | 0.0237 | *±0.0153* |
| **AMP** | 0.0358 | *±0.0120* | 0.0885 | *±0.0447* | 0.0967 | *±0.0072* |
| **ADP** | 1.3068 | *±0.0858* | 1.6779 | *±0.4517* | 1.5638 | *±0.3328* |
| **ATP** | 9.6994 | *±0.8357* | 9.3272 | *±1.3100* | 9.0561 | *±1.3941* |
| **GTP** | 2.2964 | *±0.1665* | 2.1807 | *±0.2024* | 2.1540 | *±0.3935* |
| **IMP** | n.d. |  | n.d. |  | n.d. |  |
| **Inosine** | n.d. |  | n.d. |  | n.d. |  |
